# CYP4 subfamily V member 2 (*CYP4V2*) polymorphisms were associated with ischemic stroke in Chinese Han population

**DOI:** 10.1186/s12920-022-01393-8

**Published:** 2022-11-28

**Authors:** Faqing Long, Desheng Wang, Qingjie Su, Yuhui Zhang, Jianhong Li, Shiliang Xia, Hailun Wang, Yongping Wu, Qiumin Qu

**Affiliations:** 1 Department of Neurology, The First Affiliated Hospital of Xi’an Jiaotong University, 277 West Yanta Rd, Xi’an, 710061 China; 2 Department of Neurology, The Second Affiliated Hospital of Hainan Medical University, Haikou, China; 3 Center for Brain Science, The First Affiliated Hospital of Xi’an Jiaotong University, Xi’an, China

**Keywords:** Ischemic stroke, *CYP4V2* genetic polymorphisms, Susceptibility, Confounding factors, MDR analysis

## Abstract

**Background:**

*CYP4 subfamily V member 2* (*CYP4V2*) polymorphisms are related to venous thromboembolism. However, the influence of *CYP4V2* polymorphisms on the susceptibility to ischemic stroke (IS) remains undetermined.

**Methods:**

We selected and genotyped five polymorphisms of *CYP4V2* in 575 cases and 575 controls to test whether *CYP4V2* variants were associated with the risk for IS in a Chinese Han population. Genotyping of *CYP4V2* polymorphisms was performed using the Agena MassARRAY platform. Logistic regression analysis was used to assess the association between *CYP4V2* polymorphisms and IS risk by calculating odds ratios (ORs) and 95% confidence interval (CI). False-positive report probability analysis was applied to assess the noteworthy relationship of the significant findings.

**Results:**

*CYP4V2* rs1398007 might be a risk factor for IS (OR = 1.34, 95% CI 1.05–1.71, *p* = 0.009). Specially, confounding factors (age, gender, smoking and drinking status) might affect the relationship between rs1398007 and IS susceptibility. Moreover, rs1053094 and rs56413992 were associated with IS risk in males. Multifactor dimensionality reduction analysis showed the combination of rs13146272 and rs3736455 had the strongest interaction effect (information gain value of 0.40%). Furthermore, genotypes of rs1398007 (*p* = 0.006) and rs1053094 (*p* = 0.044) were associated with the levels of high-density lipoprotein cholesterol (HDL-C) among healthy controls.

**Conclusion:**

Our results first provided evidence that *CYP4V2* rs1398007 might be a risk factor for IS, which provides instructive clues for studying the mechanisms of *CYP4V2* to the pathogenesis of IS.

**Supplementary Information:**

The online version contains supplementary material available at 10.1186/s12920-022-01393-8.

## Introduction

Stroke is the second leading cause of death worldwide and the highest mortality and disability rate in China [1]. Ischemic stroke (IS) is a severely disabling cerebrovascular disease, caused by blockage of an artery in the brain. Being one of the most common types of cerebrovascular events in China, IS accounts for 70% of all strokes [2]. Stroke can happen at any time but is more likely to occur at an older age. Prevalence rates of stroke increase with age [3]. Racial and ethnic differences also influence the progression of stroke [4]. Age-specific stroke rates are higher in men than in women [5]. Hypertension, diabetes, dyslipidaemia, smoking and alcohol consumption are the most common modifiable risk factors for IS in China [6]. Additionally, increasing evidence suggests that genetic factors may play an important role in the occurrence and etiology of IS. Genome-wide association studies (GWAS) have identified several genetic variants associated with IS risk, including *GPX7*, *LBH*, *ZCCHC10*, *DENND2A*, and *NUDT14 *[7–9]. However, many variants remain to be discovered.

Cytochrome P450 family 4 (CYP4) enzymes are related to fatty acids metabolism, which is responsible for eliminating excess free fatty acids from the body [10]. CYP4 expression is involved in angiogenesis through the production of 20-HETE [11]. Several reports have displayed that *CYP4* family genes are associated with multiple cardiovascular diseases by producing 20-HETE or interfering with fatty acid metabolism [12, 13]. CYP4 subfamily V member 2 (*CYP4V2*) is located on chromosome 4q35 and encodes a protein belonging to the P450 heme-thiolate protein superfamily [14]. *CYP4V2* was first discovered in inflammatory macrophages [15], and has been reported to be involved in fatty acid and corticosteroid metabolism [16]. A meta-analysis has demonstrated that *CYP4V2* genetic variants are the risk factors for venous thromboembolism [17]. Most stroke-related deaths are caused by thrombotic occlusion of cerebral vessels [18]. However, there are no studies to our knowledge of the effect of *CYP4V2* polymorphisms on IS susceptibility.

Here, five polymorphisms in *CYP4V2* were selected and genotyped to evaluate their impact on the susceptibility to IS in a Chinese Han population. Moreover, heterogeneity for genetic association defined by age, gender, smoking, drinking status and hypertension was investigated, and the relationship between these selected polymorphisms and clinical index was assessed.

## Subjects and methods

### Study population

All subjects were recruited from the Second Affiliated Hospital of Hainan Medical University from January 2020 to March 2021, including 575 IS cases (347 males and 228 females, 63.87 ± 10.44 years) and 575 healthy controls (344 males and 231 females, 63.09 ± 7.44 years). IS patients were diagnosed and confirmed through neurological examinations including brain computed tomography (CT) and magnetic resonance imaging (MRI) scans by two independent neurologists. Patients with brain trauma, embolic brain infarction, subarachnoid hemorrhage, other brain disease, cardiovascular diseases, tumors or serious chronic diseases were excluded. Controls were age-, gender-, race-, and geographical- matched and were from the health checkup center of the same hospital during the same period. Controls with a history of stroke, other neurological diseases, cerebrovascular, arterial vascular, and cardiovascular diseases, or inflammatory disorders were excluded. The research protocol complied with the Declaration of Helsinki and was approved by the ethics committee of the Second Affiliated Hospital of Hainan Medical University (NO. LW2021056). All subjects provided written informed consent. Demographic (age, gender, smoking and alcohol consumption) and blood biochemical indicators [total protein, serum uric acid, triglyceride total cholesterol, high-density lipoprotein cholesterol (HDL-C), low-density lipoprotein cholesterol (LDL-C), leukocyte, red blood cell (RBC), hemoglobin, and platelet] were collected from standardized questionnaires and medical records of each subject by trained research staff, respectively. For smoking, participants were classified as nonsmokers (never) and smokers (ever or current). Subjects who smoked one cigarette a day were regarded as current smokers. For drinking, participants were classified as nondrinkers (never) and drinkers (ever or current). Subjects who drank at least 100 g of alcohol a week were considered drinkers. Hypertension was determined as systolic blood pressure (SBP) ≥ 140 mmHg and/or diastolic blood pressure (DBP) ≥ 90 mmHg, or currently receiving anti-hypertensive medication.

### Laboratory investigation

Peripheral blood samples (5 mL) were collected from all participants after 12 h of fasting to perform biochemical assays on the second day of hospitalization. Plasma was obtained by centrifugation at 3000× g for 10 min. Total protein, serum uric acid, triglyceride total cholesterol, HDL-C, LDL-C, and hemoglobin were measured by a fully automatic biochemical analyzer (Hitachi 7600). Leukocyte, RBC, and platelet were detected by automatic blood cell analyzer (Beckman DxH800).

### Genotyping of CYP4V2 polymorphisms

Hardy–Weinberg equilibrium (HWE) is a general and far-reaching principle in population genetics, which has a wide range of applications. HWE is commonly used to detect genotyping errors in genetic association studies [19]. The genotypes of all single nucleotide polymorphisms (SNPs) were consistent with HWE (*p* > 0.05), which indicated the subjects had a representative of population. We selected tagSNPs based on HWE > 0.05, minor allele frequency (MAF) > 0.1, min genotype > 75%, and r^2^ > 0.8 using the e!GRCh37 database. Combined MassARRAY primer design software, MAF > 0.5 and the call rate > 95% among our study population, five functional SNPs in *CYP4V2* including rs1398007, rs13146272, rs3736455, rs1053094 and rs56413992 were selected. Bioinformatics tools including SNPinfo Web Server (https://snpinfo.niehs.nih.gov/snpinfo/index.html) and HaploReg v4.1 (https://pubs.broadinstitute.org/mammals/haploreg/haploreg.php) were used to identify the potential functional SNPs in the human *CYP4V2* gene. Blood samples (5 mL) were collected in EDTA-containing tubes, and genomic DNA was purified using commercially available DNA extraction kits (GoldMag Co. Ltd, Xi′an, China).

The MassARRAY platform is based on MALDI-TOF (matrix-assisted laser desorption/ionization—time of flight) mass spectrometry in a high-throughput and cost-effective manner [20, 21]. The general principle of the MassARRAY platform is to resolve differences in primer masses due to changes in sequence such as the binding of different terminator nucleotides at the 3’-end of the primer bound adjacent to a variation site. The analytical accuracy of MALDI-TOF MS is quite high and 0.1–0.01% of the determined mass. Genotyping of *CYP4V2* polymorphisms was performed using the Agena MassARRAY platform (Agena, San Diego, CA, USA). Primer design (Additional file 1: Table S1) and data management were performed based on corresponding supporting software, including MassARRAY Nano dispenser and Agena Bioscience Typer 4.0 software. Genotyping technology included PCR multiplex assay, shrimp alkaline phosphatase treatment, single base extension, and MALDI-TOF mass. In addition, this study also set double wells for each sample to ensure the accuracy of the results. About 10% of subjects were randomly selected for re-genotyping to verify genotyping quality, and the results were consistent.

### Statistical analysis

In order to ensure the accuracy and credibility of the research results, G*power 3.1.9.7 software (https://stats.idre.ucla.edu/other/gpower/) was used to estimate the sample size prior to the study. The specific parameters were set as follows: effect size d = 0.2; α error probability = 0.05; and power (1-β err prob) = 90%. This calculation generated a sample of at least 429 cases and 429 controls. In our study, we recruited 575 cases and 575 controls, which was larger than the total sample size recommended by G*power.

Categorical variables and continuous variables between IS patients and healthy controls were compared using the chi-square and Student’s t tests, respectively. HWE for genotype frequencies was tested by χ^2^ test. Logistic regression analysis with adjustment for age, sex, smoking, and drinking was used to calculate odds ratios (ORs) and 95% confidence intervals (CIs) to evaluate the relationship of *CYP4V2* variants to IS susceptibility. False-positive report probability (FPRP) analysis was applied to assess the noteworthy relationship of the significant findings. The threshold of FPRP was set at 0.2 for the significant relationship under investigation. Multifactor dimensionality reduction (MDR) was used to assess the optimal interaction effect of *CYP4V2* polymorphisms on IS susceptibility. Analysis of variance (ANOVA) was performed to analyze the correlation of *CYP4V2* variants with clinical features of IS patients and healthy controls. Data analysis was performed using IBM SPSS v18.0 software (Chicago, IL, USA). A two-tailed *p* value < 0.05 indicated statistical significance, and a Bonferroni-corrected *p* < 0.05/5 was considered significant.

## Results

### Characteristics of participants

Basic features of subjects were shown in Table 1. The participants consisted of 575 IS cases and 575 controls. No statistically significant differences in the distribution of age (*p* = 0.146), gender (*p* = 0.857), smoking (*p* = 0.813), and alcohol consumption (*p* = 1.000) were found. Nevertheless, differences in clinical biochemical indexes including total protein, serum uric acid, total cholesterol, HDL-C, LDL-C, leukocyte, RBC, hemoglobin, and platelet were observed between IS cases and controls (*p* < 0.001).

**Table 1:** Characteristics of IS patients and controls

Variable	Cases	Control	*p*
N	575	575	
Age (year, mean ± SD)	63.87 ± 10.44	63.09 ± 7.44	0.146
> 60	359 (62.4%)	399 (69.4%)	
≤ 60	216 (37.6%)	176 (30.6%)	
Gender			
Males	347 (60.3%)	344 (59.8%)	0.857
Females	228 (39.7%)	231 (40.2%)	
Smoking			
No	283 (49.2%)	287 (49.9%)	0.813
Yes	292 (50.8%)	288 (50.1%)	
Alcohol consumption			
No	281 (48.9%)	281 (48.9%)	1.000
Yes	294 (51.1%)	294 (51.1%)	
Total protein (g/L)	66.18 ± 5.6	71.78 ± 4.05	**< 0.0001**
Serum uric acid (µmol/L)	267.62 ± 85.55	319.13 ± 72.92	**< 0.0001**
Triglyceride (mmol/L)	1.54 ± 0.91	1.63 ± 0.67	0.055
Total cholesterol (mmol/L)	3.94 ± 0.87	4.73 ± 0.88	**< 0.0001**
HDL-C (mmol/L)	1.17 ± 0.26	1.25 ± 0.26	**< 0.0001**
LDL-C (mmol/L)	2.08 ± 0.64	2.58 ± 0.62	**< 0.0001**
Leukocyte (10^9^/L)	8.07 ± 5.57	5.77 ± 1.37	**< 0.0001**
RBC (10^9^/L)	4.50 ± 0.69	4.77 ± 0.43	**< 0.0001**
Hemoglobin (g/L)	132.82 ± 20.88	145.64 ± 13.93	**< 0.0001**
Platelet (10^9^/L)	191.6 ± 63.14	213.61 ± 56.22	**< 0.0001**
Hypertension			
No	179		
Yes	396		

*IS*, ischemic stroke;* HDL-C*, high-density lipoprotein cholesterol;* LDL-C*, low-density lipoprotein cholesterol;* RBC*, red blood cell

*p* values were calculated by χ^2^ test or Student’s t test

Bold indicate that *p* < 0.05 indicates statistical significance

### Association of CYP4V2 SNPs with IS susceptibility

Five SNPs (rs1398007, rs13146272, rs3736455, rs1053094 and rs56413992) in *CYP4V2* were selected and were consistent with HWE in the control group (*p* > 0.05, Additional file 1: Table S2). The MAFs of all SNPs in patients and controls were > 5%, suggesting that the subjects had a representative of population. The results of genotyping displayed that genotyping success rate of each SNP was > 99.5%. The allele frequencies of these *CYP4V2* SNPs were not significantly different between cases and controls (*p* > 0.05).

Additional file 1: Table S3 showed the potential functions and the MAFs of these polymorphisms in different populations. Through HaploReg annotation, these variants were found to be related to the regulation of promoter and/or enhancer histone marks, DNase, proteins bound, motifs changed, GRASP quantitative trait locus (QTL) hits and/or selected eQTL hits. The SNPinfo web server database displayed that rs1398007 might be a transcription factor binding sites (TFBS), and rs13146272 and rs3736455 might be associated with splicing. Moreover, rs1053094 and rs56413992 located in the 3’-UTR region of the *CYP4V2* gene might be related to the binding of miRNA.

The association of *CYP4V2* SNPs with IS susceptibility was assessed (Table 2). *CYP4V2* rs1398007 might be a risk factor for IS under the codominant (OR = 1.34, 95% CI 1.05–1.71, *p* = 0.009) model. The significant association between *CYP4V2* rs1398007 and IS risk still existed after Bonferroni correction (*p* < 0.05/5).

**Table 2:** Association between *CYP4V2* polymorphisms and IS risk

SNP ID	Model	Genotype	Control	Case	OR (95% CI)	P-value	AIC	BIC
rs1398007	Codominant	C/C	272 (47.3%)	242 (42.1%)	1	**0.009**	1596.7	1632
		T/C	250 (43.5%)	298 (51.8%)	**1.34 (1.05–1.71)**			
		T/T	53 (9.2%)	35 (6.1%)	0.76 (0.48–1.20)			
	Dominant	C/C	272 (47.3%)	242 (42.1%)	1	0.073	1600.7	1631
		T/C-T/T	303 (52.7%)	333 (57.9%)	1.24 (0.98–1.56)			
	Log-additive	–	–	–	1.06 (0.88–1.28)	0.530	1603.6	1633.9
rs13146272	Codominant	C/C	228 (39.8%)	229 (39.8%)	1	0.700	1602.4	1637.7
		C/A	268 (46.8%)	259 (45%)	0.96 (0.74–1.23)			
		A/A	77 (13.4%)	87 (15.1%)	1.11 (0.78–1.59)			
	Dominant	C/C	228 (39.8%)	229 (39.8%)	1	0.950	1601.1	1631.4
		C/A-A/A	345 (60.2%)	346 (60.2%)	0.99 (0.78–1.26)			
	Log-additive	–	–	–	1.03 (0.87–1.22)	0.730	1601	1631.3
rs3736455	Codominant	T/T	207 (36.1%)	201 (35%)	1	0.400	1601.4	1636.7
		G/T	292 (51%)	283 (49.2%)	1.00 (0.77–1.29)			
		G/G	74 (12.9%)	91 (15.8%)	1.26 (0.87–1.81)			
	Dominant	T/T	207 (36.1%)	201 (35%)	1	0.700	1601	1631.3
		G/T-G/G	366 (63.9%)	374 (65%)	1.05 (0.82–1.34)			
	Log-additive	–	–	–	1.09 (0.92–1.29)	0.330	1600.2	1630.5
rs1053094	Codominant	T/T	245 (42.8%)	228 (39.6%)	1	0.530	1602	1637.3
		T/A	263 (45.9%)	278 (48.4%)	1.14 (0.89–1.46)			
		A/A	65 (11.3%)	69 (12%)	1.16 (0.79–1.71)			
	Dominant	T/T	245 (42.8%)	228 (39.6%)	1	0.260	1600	1630.2
		T/A-A/A	328 (57.2%)	347 (60.4%)	1.14 (0.90–1.45)			
	Log-additive	–	–	–	1.10 (0.92–1.31)	0.300	1600.1	1630.4
rs56413992	Codominant	C/C	369 (64.3%)	359 (62.5%)	1	0.780	1602.5	1637.9
		T/C	182 (31.7%)	190 (33.1%)	1.07 (0.83–1.38)			
		T/T	23 (4%)	25 (4.4%)	1.17 (0.65–2.11)			
	Dominant	C/C	369 (64.3%)	359 (62.5%)	1	0.520	1600.6	1630.9
		T/C-T/T	205 (35.7%)	215 (37.5%)	1.08 (0.85–1.38)			
	Log-additive	–	–	–	1.08 (0.88–1.32)	0.480	1600.5	1630.8

IS, ischemic stroke;* SNP*, single nucleotide polymorphism;* OR*, odds ratio;* 95% CI*, 95% confidence interval;* AIC*, akaike information criterion;* BIC*, bayesian information criterion

*p* values were calculated using logistic regression analysis adjusted by gender, age, smoking and drinking

Bold indicate that *p* < 0.05 means the data is statistically significant

### Stratification analysis for the genetic correlation

The contribution of confounding factors (age, gender, smoking and drinking status) to the genetic association between *CYP4V2* polymorphisms and IS susceptibility was evaluated (Table 3). Stratified analysis by age, comparable risk effect against IS was observed for rs1398007 under the codominant (OR = 1.64, 95% CI 1.06–2.56, *p* = 0.028) model in subjects aged ≤ 60 years.

**Table 3:** Association between *CYP4V2* polymorphisms and IS risk according to the stratification analysis

SNP ID	Model	Genotype	Control	Case	OR (95% CI)	*p*-value	Control	Case	OR (95% CI)	*p*-value
Age, years		> 60					≤ 60			
rs1398007	Codominant	C/C	182 (45.6%)	152 (42.3%)	1	0.240	90 (51.1%)	90 (41.7%)	1	**0.028**
		T/C	182 (45.6%)	188 (52.4%)	1.21 (0.88–1.65)		68 (38.6%)	110 (50.9%)	**1.64 (1.06–2.56)**	
		T/T	35 (8.8%)	19 (5.3%)	0.76 (0.41–1.42)		18 (10.2%)	16 (7.4%)	0.83 (0.39–1.80)	
	Dominant	C/C	182 (45.6%)	152 (42.3%)	1	0.400	90 (51.1%)	90 (41.7%)	1	0.074
		T/C-T/T	217 (54.4%)	207 (57.7%)	1.14 (0.84–1.54)		86 (48.9%)	126 (58.3%)	1.47 (0.96–2.23)	
	Log-additive	–	–	–	1.02 (0.80–1.31)	0.850	–	–	1.16 (0.84–1.61)	0.370
Gender		Males					Females			
rs1398007	Codominant	C/C	165 (48%)	147 (42.4%)	1	**0.040**	107 (46.3%)	95 (41.7%)	1	0.109
		T/C	151 (43.9%)	178 (51.3%)	**1.37 (1.01–1.88)**		99 (42.9%)	120 (52.6%)	1.37 (0.93–2.02)	
		T/T	28 (8.1%)	22 (6.3%)	0.90 (0.49–1.64)		25 (10.8%)	13 (5.7%)	0.57 (0.27–1.18)	
	Dominant	C/C	165 (48%)	147 (42.4%)	1	0.095	107 (46.3%)	95 (41.7%)	1	0.320
		T/C-T/T	179 (52%)	200 (57.6%)	1.29 (0.96–1.75)		124 (53.7%)	133 (58.3%)	1.21 (0.83–1.75)	
	Log-additive	–	–	–	1.13 (0.88–1.44)	0.330	–	–	0.98 (0.73–1.32)	0.910
rs1053094	Codominant	T/T	152 (44.2%)	124 (35.7%)	1	**0.029**	93 (40.6%)	104 (45.6%)	1	0.370
		T/A	160 (46.5%)	177 (51%)	1.33 (0.96–1.83)		103 (45%)	101 (44.3%)	0.87 (0.59–1.29)	
		A/A	32 (9.3%)	46 (13.3%)	**1.80 (1.08-3.00)**		33 (14.4%)	23 (10.1%)	0.65 (0.35–1.19)	
	Dominant	T/T	152 (44.2%)	124 (35.7%)	1	**0.030**	93 (40.6%)	104 (45.6%)	1	0.290
		T/A-A/A	192 (55.8%)	223 (64.3%)	**1.40 (1.03–1.91)**		136 (59.4%)	124 (54.4%)	0.82 (0.56–1.19)	
	Log-additive	–	–	–	**1.34 (1.06–1.68)**	**0.014**	–	–	0.83 (0.63–1.09)	0.170
rs56413992	Codominant	C/C	230 (66.9%)	208 (59.9%)	1	0.130	139 (60.4%)	151 (66.5%)	1	0.410
		T/C	106 (30.8%)	127 (36.6%)	1.29 (0.94–1.78)		76 (33%)	63 (27.8%)	0.76 (0.50–1.15)	
		T/T	8 (2.3%)	12 (3.5%)	1.97 (0.78–4.97)		15 (6.5%)	13 (5.7%)	0.85 (0.39–1.87)	
	Dominant	C/C	230 (66.9%)	208 (59.9%)	1	0.068	139 (60.4%)	151 (66.5%)	1	0.190
		T/C-T/T	114 (33.1%)	139 (40.1%)	1.34 (0.98–1.83)		91 (39.6%)	76 (33.5%)	0.77 (0.53–1.14)	
	Log-additive	–	–	–	**1.33 (1.01–1.75)**	**0.045**	–	–	0.84 (0.62–1.14)	0.260
Smoking			Smokers				Non-smokers			
rs1398007	Codominant	C/C	142 (49.3%)	133 (45.5%)	1	0.200	130 (45.3%)	109 (38.5%)	1	**0.010**
		T/C	120 (41.7%)	140 (48%)	1.30 (0.92–1.85)		130 (45.3%)	158 (55.8%)	**1.51 (1.06–2.14)**	
		T/T	26 (9%)	19 (6.5%)	0.83 (0.43–1.60)		27 (9.4%)	16 (5.7%)	0.65 (0.33–1.28)	
	Dominant	C/C	142 (49.3%)	133 (45.5%)	1	0.240	130 (45.3%)	109 (38.5%)	1	0.084
		T/C-T/T	146 (50.7%)	159 (54.5%)	1.22 (0.87–1.70)		157 (54.7%)	174 (61.5%)	1.35 (0.96–1.89)	
	Log-additive	–	–	–	1.07 (0.82–1.40)	0.600	–	–	1.08 (0.82–1.41)	0.59
Drinking			Drinkers				Non-drinkers			
rs1398007	Codominant	C/C	137 (46.6%)	129 (43.9%)	1	0.700	135 (48%)	113 (40.2%)	1	**0.004**
		T/C	130 (44.2%)	142 (48.3%)	1.14 (0.80–1.60)		120 (42.7%)	156 (55.5%)	**1.66 (1.17–2.37)**	
		T/T	27 (9.2%)	23 (7.8%)	0.93 (0.50–1.74)		26 (9.2%)	12 (4.3%)	0.53 (0.25–1.10)	
	Dominant	C/C	137 (46.6%)	129 (43.9%)	1	0.560	135 (48%)	113 (40.2%)	1	**0.026**
		T/C-T/T	157 (53.4%)	165 (56.1%)	1.10 (0.79–1.54)		146 (52%)	168 (59.8%)	**1.45 (1.03–2.03)**	
	Log-additive	–	–	–	1.04 (0.80–1.34)	0.800	–	–	1.10 (0.84–1.45)	0.490

*IS*, ischemic stroke;* SNP*, single nucleotide polymorphism;* OR*, odds ratio;* 95% CI*, 95% confidence interval

*p* values were calculated using logistic regression analysis adjusted by gender, age, smoking and/or drinking

Bold indicate that *p* < 0.05 means the data is statistically significant

In the stratified analysis by gender, rs1398007 (OR = 1.37, 95% CI 1.01–1.88, *p* = 0.040), rs1053094 (codominant: OR = 1.80, 95% CI 1.08–3.00, *p* = 0.029; dominant OR = 1.40, 95% CI 1.03–1.91, *p* = 0.030; log-additive: OR = 1.34, 95% CI 1.06–1.68, *p* = 0.014) and rs56413992 (log-additive: OR = 1.33, 95% CI 1.01–1.75, *p* = 0.045) were observed to be significant genetic risk variants for IS in males. In addition, rs1398007 was also associated with the development of IS (OR = 1.50, 95% CI 1.03–2.17, *p* = 0.033) among females.

In the stratified analysis by smoking and drinking status, we found that rs1398007 was associated with increased susceptibility to IS among non-smokers (OR = 1.51, 95% CI 1.06–2.14, *p* = 0.010) or non-drinkers (codominant: OR = 1.66, 95% CI 1.17–2.37, *p* = 0.004; dominant: OR = 1.45, 95% CI 1.03–2.03, *p* = 0.026), respectively. However, no significant correlation of these polymorphisms with IS risk in smokers and drinkers was found.

### Association between CYP4V2 rs1398007 and IS patients with hypertension

The relationship between *CYP4V2* SNPs and IS risk in IS patients with or without hypertension compared with healthy controls was also assessed (Table 4). Compared with healthy controls, *CYP4V2* rs1398007 was associated with an increased risk for IS patients with hypertension (OR = 1.50, 95% CI 1.14–1.96, *p* = 0.008; OR = 1.40, 95% CI 1.08–1.82, *p* = 0.010), while it exerted a protective effect on IS patients without hypertension (OR = 0.37, 95% CI 0.15–0.88, *p* = 0.031). Genotype frequencies between IS patients with hypertension and IS patients without hypertension were compared, *CYP4V2* rs1398007 (codominant: OR = 2.63, 95% CI 1.05–6.61, *p* = 0.035; dominant OR = 1.49, 95% CI 1.04–2.13, *p* = 0.030; log-additive: OR = 1.48, 95% CI 1.09–2.02, *p* = 0.011) was observed to be a genetic risk variant for IS patients with hypertension.

**Table 4:** Association between* CYP4V2* rs1398007 and IS patients with and without hypertension

Model	Genotype	Control	IS with HYP	IS without HYP	IS patients with HYP vs. controls		IS patients without HYP vs. controls		IS patients with HYP vs. without HYP	
Codominant	C/C	272 (47.3%)	155 (39.1%)	87 (48.6%)	1	**0.008**	1	**0.031**	1	**0.035**
	T/C	250 (43.5%)	212 (53.5%)	86 (48%)	**1.50 (1.14–1.96)**		1.05 (0.74–1.48)		1.41 (0.98–2.03)	
	T/T	53 (9.2%)	29 (7.3%)	6 (3.4%)	0.97 (0.59–1.59)		**0.37 (0.15–0.88)**		**2.63 (1.05–6.61)**	
Dominant	C/C	272 (47.3%)	155 (39.1%)	87 (48.6%)	1	**0.010**	1	0.680	1	**0.030**
	T/C-T/T	303 (52.7%)	241 (60.9%)	92 (51.4%)	**1.40 (1.08–1.82)**		0.93 (0.66–1.31)		**1.49 (1.04–2.13)**	
Log-additive	–	–	–	–	1.18 (0.96–1.44)	0.120	0.83 (0.63–1.09)	0.170	**1.48 (1.09–2.02)**	**0.011**

*IS*, ischemic stroke;* HYP*, hypertension;* SNP*, single nucleotide polymorphism;* OR*, odds ratio;* 95% CI*, 95% confidence interval

*p* values were calculated using logistic regression analysis adjusted by gender, age, smoking and/or drinking

Bold indicate that *p* < 0.05 means the data is statistically significant

### FPRP analysis for the association of CYP4V2 SNPs with IS susceptibility

FPRP analysis was performed to interrogate whether the important findings were worthy of attention (Table 5). At a prior probability level of 0.1, the significant relationship between rs1398007 and IS susceptibility (FPRP = 0.170) was still noteworthy in the overall analysis. The significant finding for rs1053094 (FPRP = 0.108) remained noteworthy among males. Moreover, the relationship between rs1398007 and IS risk in non-smokers (FPRP = 0.164) or non-drinkers (FPRP = 0.053) was also significant at the prior probability level of 0.1. This significant relationship between rs1398007 and the risk of IS patients with hypertension was still noteworthy (vs. controls: FPRP = 0.026 and FPRP = 0.134; vs. patients without hypertension: FPRP = 0.111).

**Table 5:** False-positive report probability values for the associations between *CYP4V2* polymorphisms and IS susceptibility

SNP ID	OR (95% CI)	*p*	Statistical power	Prior probability				
				0.25	0.1	0.01	0.001	0.0001
*Overall *								
rs1398007	1.34 (1.05–1.71)	0.009	0.818	**0.064**	**0.170**	0.693	0.958	0.996
*Age < 60 years *								
rs1398007	1.64 (1.06–2.56)	0.028	0.809	**0.098**	0.247	0.783	0.973	0.997
*Males*								
rs1398007	1.37 (1.01–1.88)	0.040	0.686	**0.177**	0.393	0.877	0.986	0.999
rs1053094	1.80 (1.08-3.00)	0.029	0.657	**0.099**	0.248	0.784	0.973	0.997
	1.40 (1.03–1.91)	0.030	0.668	**0.132**	0.312	0.833	0.981	0.998
	1.34 (1.06–1.68)	0.014	0.836	**0.039**	**0.108**	0.570	0.930	0.993
rs56413992	1.33 (1.01–1.75)	0.045	0.805	**0.134**	0.318	0.837	0.981	0.998
*Non-smokers *								
rs1398007	1.51 (1.06–2.14)	0.010	0.943	**0.061**	**0.164**	0.683	0.956	0.995
*Non-drinkers*								
rs1398007	1.66 (1.17–2.37)	0.004	0.847	**0.018**	**0.053**	0.381	0.861	0.984
	1.45 (1.03–2.03)	0.026	0.578	**0.136**	0.321	0.839	0.981	0.998
*IS patients with HYP vs. Controls *								
rs1398007	1.50 (1.14–1.96)	0.008	0.982	**0.009**	**0.026**	0.230	0.751	0.968
	1.40 (1.08–1.82)	0.010	0.697	**0.049**	**0.134**	0.629	0.945	0.994
*IS patients without HYP vs. Controls *								
rs1398007	0.37 (0.15–0.88)	0.031	0.248	0.229	0.471	0.907	0.990	0.999
*IS patients with HYP vs. without HYP*								
rs1398007	2.63 (1.05–6.61)	0.035	0.280	0.298	0.561	0.934	0.993	0.999
	1.49 (1.04–2.13)	0.030	0.515	**0.083**	0.214	0.750	0.968	0.997
	1.48 (1.09–2.02)	0.011	0.971	**0.040**	**0.111**	0.579	0.933	0.993

Bold indicate that level of false-positive report probability < 0.2

Statistical power was calculated using the number of observations in the subgroup and the OR and *p* values in this table. The level of false-positive report probability threshold was set at 0.2, and noteworthy findings are presented

### MDR analysis for SNP-SNP interaction

**Fig. 1:**
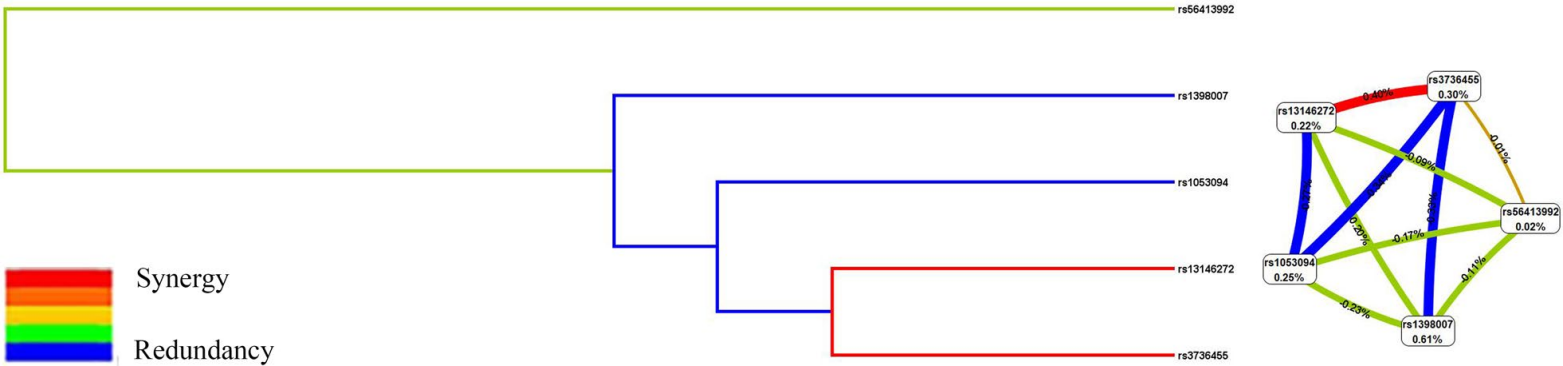
Dendrogram (left) and the Fruchterman-Reingold (right) for the interactions between these SNPs. Positive percent entropy indicates synergy whereas the negative percent indicates redundancy. Orange line indicated positive interaction, green and blue color indicated weak interactions

The interactions of these SNPs in *CYP4V2* were assessed using MDR analysis, and the results were demonstrated in Table 6. *CYP4V2* rs1398007 was the best single-locus model for predicting IS susceptibility (testing accuracy, 0.5417; *p* = 0.0046; cross-validation consistency: 10/10). The best multi-locus model was the two-locus model, the combination of rs13146272 and rs3736455, with the highest testing accuracy (0.5139). As shown in Fig. 1, the dendrogram and Fruchterman-Reingold plot displayed the interactions between these SNPs. The entropy patterns summarized the main and/or interaction effect of each paired attribute combination. The strongest interaction was the interaction between rs13146272 and rs3736455 with the information gain value of 0.40%.

**Table 6:** MDR analysis for SNP–SNP interaction in *CYP4V2* with IS susceptibility

Model	Training bal. acc.	Testing bal. acc.	CVC	OR (95% CI)	*p*
rs1398007	0.5417	0.5417	10/10	1.40 (1.11–1.76)	**0.0046**
rs13146272, rs3736455	0.5503	0.5139	5/10	1.71 (1.29–2.25)	**0.0001**
rs1398007, rs3736455, rs1053094	0.5638	0.5104	7/10	1.65 (1.31–2.09)	**< 0.0001**
rs1398007, rs13146272, rs3736455, rs1053094	0.5818	0.5130	10/10	1.96 (1.54–2.49)	**< 0.0001**
rs1398007, rs13146272, rs3736455, rs1053094, rs56413992	0.5940	0.5000	10/10	2.07 (1.63–2.63)	**< 0.0001**

*MDR*, multifactor dimensionality reduction;* SNP*, single nucleotide polymorphism;* Bal. Acc.*, balanced accuracy;* CVC*, cross–validation consistency;* OR*, odds ratio;* CI*, confidence interval

*p* values were calculated using χ^2^ tests

Bold indicate that *p* < 0.05 indicates statistical significance

### The relationship of CYP4V2 SNPs with clinical characteristics among IS patients/healthy controls

The relationship of *CYP4V2* SNPs with clinical characteristics among IS patients/healthy controls was assessed (Table 7). The genotypes of rs1398007 (*p* = 0.006) and rs1053094 (*p* = 0.044) were associated with the levels of HDL-C among healthy controls. Moreover, the genotypes of rs13146272 was related with the levels of RBC in IS patients.

**Table 7:** Association of clinical characteristics with different genotypes of *CYP4V2* polymorphisms among IS patients

Characteristics	Control				Case			
	rs1398007				rs1398007			
	TT	TC	CC	*p*	TT	TC	CC	*p*
Total protein (g/L)	72.54 ± 3.16	71.67 ± 4.01	71.73 ± 4.23	0.351	66.63 ± 6.19	66.1 0 ± 5.58	66.20 ± 5.56	0.871
Serum uric acid (mmol/L)	308.74 ± 61.7	316.42 ± 74.75	323.65 ± 73.14	0.292	287.74 ± 77.75	265.63 ± 87.92	267.15 ± 83.6	0.350
Triglyceride (mmol/L)	1.63 ± 0.74	1.60 ± 0.62	1.65 ± 0.71	0.662	1.48 ± 0.86	1.54 ± 0.89	1.54 ± 0.93	0.931
Total cholesterol (mmol/L)	4.69 ± 0.95	4.79 ± 0.87	4.69 ± 0.88	0.441	4.00 ± 0.85	3.98 ± 0.89	3.88 ± 0.85	0.341
HDL-C (mmol/L)	1.24 ± 0.27	1.29 ± 0.26	1.21 ± 0.25	**0.006**	1.15 ± 0.28	1.16 ± 0.23	1.18 ± 0.29	0.535
LDL-C (mmol/L)	2.50 ± 0.69	2.60 ± 0.61	2.57 ± 0.61	0.566	2.06 ± 0.56	2.06 ± 0.63	2.10 ± 0.66	0.791
Leukocyte (10^9^/L)	5.46 ± 1.32	5.81 ± 1.42	5.79 ± 1.34	0.224	8.31 ± 3.67	8.17 ± 6.16	7.92 ± 5.02	0.845
RBC (10^9^/L, IQR)	4.80 ± 0.46	4.77 ± 0.44	4.76 ± 0.42	0.814	4.68 ± 0.64	4.52 ± 0.65	4.45 ± 0.74	0.145
Hemoglobin (g/L)	147.17 ± 13.25	145.28 ± 13.58	145.68 ± 14.4	0.667	137 ± 18.32	132.88 ± 20.84	132.14 ± 21.29	0.437
Platelet (10^9^/L)	209.68 ± 55.98	210.22 ± 53.03	217.5 ± 58.99	0.291	183.11 ± 58.84	194.94 ± 66.56	188.71 ± 59.27	0.374

Characteristics	Control				Case			
	rs13146272				rs13146272			
	AA	CA	CC	*p*	AA	CA	CC	*p*
Total protein (g/L)	72.11 ± 3.57	71.65 ± 3.69	71.85 ± 4.57	0.663	65.53 ± 5.15	66.31 ± 5.74	66.26 ± 5.62	0.506
Serum uric acid (mmol/L)	330.88 ± 69.03	320.11 ± 74.14	313.6 ± 72.71	0.187	254.25 ± 86.03	270.54 ± 85.26	269.38 ± 85.6	0.284
Triglyceride (mmol/L)	1.62 ± 0.69	1.67 ± 0.68	1.58 ± 0.66	0.352	1.57 ± 1.28	1.57 ± 0.90	1.48 ± 0.72	0.500
Total cholesterol (mmol/L)	4.74 ± 0.85	4.76 ± 0.91	4.71 ± 0.87	0.857	3.83 ± 0.89	4.02 ± 0.93	3.89 ± 0.78	0.101
HDL-C (mmol/L)	1.21 ± 0.25	1.24 ± 0.26	1.28 ± 0.25	0.102	1.16 ± 0.20	1.18 ± 0.29	1.15 ± 0.24	0.342
LDL-C (mmol/L)	2.57 ± 0.51	2.61 ± 0.64	2.56 ± 0.63	0.646	2.06 ± 0.66	2.13 ± 0.65	2.03 ± 0.62	0.244
Leukocyte (10^9^/L)	5.92 ± 1.35	5.83 ± 1.36	5.64 ± 1.40	0.192	7.74 ± 3.49	7.98 ± 4.45	8.31 ± 7.14	0.679
RBC (10^9^/L, IQR)	4.80 ± 0.44	4.76 ± 0.42	4.77 ± 0.44	0.800	4.46 ± 0.74	4.43 ± 0.69	4.59 ± 0.66	**0.036**
Hemoglobin (g/L)	146.39 ± 14.44	146.01 ± 13.65	145.08 ± 14.08	0.680	132.13 ± 21.00	131.27 ± 20.76	134.85 ± 20.9	0.158
Platelet (10^9^/L)	223.65 ± 62.88	214.16 ± 52.87	209.64 ± 57.69	0.165	185.68 ± 70.62	188.01 ± 61.23	197.91 ± 62.00	0.143

Characteristics	Control				Case			
	rs3736455				rs3736455			
	TT	GT	GG	*p*	TT	GT	GG	*p*
Total protein (g/L)	71.74 ± 4.59	71.74 ± 3.76	71.97 ± 3.54	0.904	66.11 ± 5.38	66.27 ± 5.85	66.01 ± 5.34	0.910
Serum uric acid (mmol/L)	309.81 ± 73.20	324.2 ± 73.07	325.34 ± 70.81	0.070	267.19 ± 82.16	271.70 ± 86.42	255.85 ± 89.89	0.306
Triglyceride (mmol/L)	1.58 ± 0.68	1.65 ± 0.66	1.64 ± 0.74	0.547	1.50 ± 0.85	1.55 ± 0.81	1.56 ± 1.26	0.802
Total cholesterol (mmol/L)	4.73 ± 0.88	4.73 ± 0.90	4.74 ± 0.84	0.998	3.95 ± 0.74	3.96 ± 0.94	3.85 ± 0.89	0.553
HDL-C (mmol/L)	1.28 ± 0.25	1.23 ± 0.26	1.22 ± 0.26	0.100	1.16 ± 0.24	1.17 ± 0.28	1.16 ± 0.25	0.875
LDL-C (mmol/L)	2.56 ± 0.65	2.59 ± 0.63	2.57 ± 0.51	0.879	2.04 ± 0.62	2.10 ± 0.64	2.08 ± 0.68	0.604
Leukocyte (10^9^/L)	5.62 ± 1.38	5.84 ± 1.38	5.89 ± 1.33	0.166	7.98 ± 6.11	8.17 ± 5.73	8.00 ± 3.44	0.928
RBC (10^9^/L, IQR)	4.76 ± 0.43	4.77 ± 0.43	4.78 ± 0.47	0.892	4.56 ± 0.68	4.45 ± 0.68	4.49 ± 0.71	0.210
Hemoglobin (g/L)	144.78 ± 13.63	146.19 ± 14.00	145.82 ± 14.66	0.537	134.57 ± 20.37	131.89 ± 21.21	131.86 ± 20.96	0.339
Platelet (10^9^/L)	210.56 ± 57.84	213.45 ± 53.09	223.74 ± 63.33	0.223	192.76 ± 60.68	191.06 ± 58.57	190.73 ± 80.55	0.949

Characteristics	Control				Case			
	rs1053094				rs1053094			
	AA	TA	TT	*p*	TT	TA	TT	*p*
Total protein (g/L)	72.07 ± 3.78	71.9 ± 4.25	71.57 ± 3.91	0.557	67.18 ± 5.16	66.14 ± 5.54	65.92 ± 5.79	0.259
Serum uric acid (mmol/L)	317.85 ± 71.96	315.02 ± 71.73	323.83 ± 74.62	0.393	276.32 ± 71.32	265.82 ± 84.53	267.18 ± 90.76	0.657
Triglyceride (mmol/L)	1.59 ± 0.62	1.68 ± 0.71	1.58 ± 0.64	0.232	1.67 ± 1.31	1.53 ± 0.83	1.50 ± 0.85	0.408
Total cholesterol (mmol/L)	4.88 ± 0.83	4.67 ± 0.90	4.76 ± 0.88	0.202	3.86 ± 0.62	3.99 ± 0.94	3.90 ± 0.84	0.350
HDL-C (mmol/L)	1.32 ± 0.22	1.23 ± 0.27	1.25 ± 0.26	**0.044**	1.15 ± 0.22	1.18 ± 0.27	1.16 ± 0.26	0.596
LDL-C (mmol/L)	2.65 ± 0.56	2.51 ± 0.63	2.63 ± 0.61	0.059	2.00 ± 0.62	2.10 ± 0.65	2.08 ± 0.63	0.492
Leukocyte (10^9^/L)	5.64 ± 1.43	5.75 ± 1.34	5.83 ± 1.39	0.582	8.28 ± 6.51	8.50 ± 6.74	7.50 ± 3.13	0.124
RBC (10^9^/L, IQR)	4.69 ± 0.41	4.79 ± 0.47	4.77 ± 0.40	0.229	4.67 ± 0.72	4.48 ± 0.67	4.47 ± 0.69	0.093
Hemoglobin (g/L)	144.51 ± 12.37	145.88 ± 14.72	145.85 ± 13.41	0.760	136.32 ± 22.25	133.03 ± 21.41	131.51 ± 19.74	0.239
Platelet (10^9^/L)	211.77 ± 44.60	213.06 ± 56.18	214.42 ± 59.08	0.931	200.97 ± 46.67	191.51 ± 69.22	188.87 ± 59.55	0.378

Characteristics	Control				Case			
	rs56413992				rs56413992			
	TT	TC	CC	*p*	TT	TC	CC	*p*
Total protein (g/L)	72.30 ± 4.02	71.68 ± 4.23	71.79 ± 3.96	0.778	67.90 ± 5.50	66.50 ± 5.47	65.89 ± 5.67	0.140
Serum uric acid (mmol/L)	300.22 ± 60.18	312.88 ± 66.40	323.40 ± 76.48	0.126	251.40 ± 80.52	271.11 ± 80.4	267.01 ± 88.65	0.541
Triglyceride (mmol/L)	1.55 ± 0.51	1.58 ± 0.67	1.66 ± 0.69	0.391	1.53 ± 0.81	1.44 ± 0.71	1.58 ± 0.98	0.254
Total cholesterol (mmol/L)	4.97 ± 0.75	4.67 ± 0.88	4.75 ± 0.89	0.273	3.74 ± 0.62	3.96 ± 0.80	3.94 ± 0.92	0.475
HDL-C (mmol/L)	1.29 ± 0.18	1.26 ± 0.25	1.24 ± 0.26	0.383	1.13 ± 0.23	1.19 ± 0.25	1.15 ± 0.27	0.164
LDL-C (mmol/L)	2.67 ± 0.56	2.51 ± 0.62	2.61 ± 0.62	0.188	1.94 ± 0.69	2.07 ± 0.63	2.09 ± 0.65	0.511
Leukocyte (10^9^/L)	5.46 ± 1.49	5.69 ± 1.40	5.82 ± 1.36	0.310	8.00 ± 2.43	8.21 ± 6.25	7.98 ± 5.34	0.901
RBC (10^9^/L, IQR)	4.68 ± 0.27	4.73 ± 0.47	4.79 ± 0.42	0.198	4.50 ± 0.63	4.56 ± 0.65	4.46 ± 0.71	0.303
Hemoglobin (g/L)	143.48 ± 9.34	144.32 ± 14.93	146.43 ± 13.64	0.184	134.44 ± 17.18	134.82 ± 20.4	131.62 ± 21.35	0.217
Platelet (10^9^/L)	210.22 ± 42.7	210.00 ± 57.29	215.61 ± 56.53	0.523	203.52 ± 48.85	192.53 ± 70.13	190.37 ± 60.16	0.588

*IS*, ischemic stroke;* HDL-C*, high-density lipoprotein cholesterol;* LDL-C*, low-density lipoprotein cholesterol;* TLC*, total leukocyte count;* PLT*, platelets

*p* values were calculated using Analysis of Variance (ANOVA).

Bold indicate that *p* < 0.05 indicates statistical significance

## Discussion

In the present study, *CYP4V2* rs1398007 might be a risk factor for IS after Bonferroni correction. Specially, confounding factors (age, gender, smoking, and drinking status) might affect the association of rs1398007 with IS susceptibility. Moreover, rs1053094 and rs56413992 were also observed to be significant genetic risk variants for IS occurrence among males. MDR analysis showed that the best multi- locus model was the two-locus model, the combination of rs13146272 and rs3736455 with the strongest interaction effect (information gain values of 0.40%). Furthermore, genotypes of rs1398007 (*p* = 0.006) and rs1053094 (*p* = 0.044) were related to the levels of HDL-C among healthy controls. Our results first revealed the relationship between *CYP4V2* variants and IS occurrence in the Chinese Han population.

In the study, five SNPs in *CYP4V2* were selected based on MAF > 0.5, HWE > 0.5 and call rate > 95%. The Hapmap project takes SNPs with MAF > 0.05 as the primary research target [22]. MAF is widely used in GWAS of complex diseases. In association studies, a smaller MAF will reduce statistical power, resulting in false negative results. HWE test is commonly used to detect genotyping errors in genetic association studies [19]. *p* > 0.05 indicates that the study population has reached a genetic balance, that is, the data of this population investigation is credible. The success rate of genotyping often reflects the quality of DNA samples and the applicability of genotyping technology, which affects the reliability of data analysis results to a certain extent. We chose SNPs with call rate greater than 95% to eliminate low-quality SNPs. In this study, SNP selection based on these SNP criteria is helpful to improve the reliability of the analysis results and to reduce the false negative/positive rate.

IS is a complicated heterogeneous multifactorial and multi-gene disease [23]. Several researches have displayed that genetic polymorphisms in CYP4 family genes such as *CYP4A11* and *CYP4F2* are related to the susceptibility to cardiovascular diseases [24, 25]. Variants in *CYP4V2* are related to the occurrence of deep vein thrombosis and Bietti corneoretinal crystalline dystrophy [26, 27]. However, there are no reports on the association of *CYP4V2* variants with IS susceptibility. Our results first displayed that *CYP4V2* rs1398007 might be a risk factor for IS after Bonferroni correction in the Chinese Han population. The mutant allele frequency of rs1398007 was quite different between different ethnic groups. The MAF of rs1398007 in Asian populations (0.29) was similar to that in African populations (0.23) and lower than that in American (0.44) and European (0.53) populations, according to the HaploReg database. These results suggested that the association of rs1398007 with IS susceptibility might be related to racial differences, and our findings need to be confirmed in various populations. In addition, there is no report on the functional mechanism of rs1398007. Based on the HaploReg database, rs1398007 might be involved in the regulation of promoter histone marks, DNAse, proteins binding, changed motifs and selected eQTL hits. Further functional assays are needed to investigate the underlying function and mechanism of rs1398007 in IS progression.

Age and sex are key factors in IS pathology [28]. The incidence of ischemic vascular disease increases with age. Compared with younger patients, elderly patients with stroke have a higher mortality rate and a worse quality of life [29, 30]. Gender also affects the prevalence and outcomes of stroke, and males have a higher incidence of stroke than females throughout most of the lifespan [31, 32]. In the stratification analysis by age, rs1398007 was correlated with increased IS risk in subjects aged ≤ 60 years, but not in subjects aged > 60 years. Stratified by sex, rs1398007, rs1053094 and rs56413992 were associated with higher IS susceptibility among males, but not among females. These results demonstrated that the effect of *CYP4V2* genetic variants on IS occurrence was age- and gender-specific.

Studies have found that cigarette smoking may increase IS risk through the content of oxides of nitrogen, free radicals and other toxic substances [33]. Additionally, a previous study has reported that light and moderate drinking is only inversely related to IS risk, while heavy alcohol consumption increases the occurrence of all stroke types [34]. Therefore, we explored the heterogeneity of smoking and drinking on the relationship between *CYP4V2* variants and IS risk. When stratified by smoking and drinking status, we found that rs1398007 was associated with increased susceptibility to IS among non-smokers or non-drinkers. Hypertension is positively associated with the risk of IS [35]. In our study, rs1398007 was also found to be associated with increased risk of IS patients with hypertension. Due to the moderate sample size after stratification, it is necessary to further verify our results in a larger sample size.

HDL-C is also considered to be a risk factor for IS [36]. We found that the genotypes of rs1398007 and rs1053094 were associated with the levels of HDL-C among healthy controls, but not in IS patients, which might be caused by abnormal HDL-C levels in IS patients. The results revealed that *CYP4V2* polymorphisms might have a potential impact on serum HDL-C concentration. Nonetheless, more functional studies are necessary.

Several potential limitations are unavoidable in our study. First, the study population was a Chinese Han population from the same hospital. Therefore, the inherent selection bias was inevitable, and our results need to be further evaluated in other ethnic groups. Second, we analyzed only five SNPs in the *CYP4V2* gene, which may not represent the whole gene, and the functional roles of *CYP4V2* polymorphisms in the pathogenesis of IS were not assessed. Third, a limitation is insufficient information about subtypes of stroke. It will be interesting to study whether *CYP4V2* rs1398007 is related to other stroke subtypes.

These findings may deepen our understanding of *CYP4V2* in the occurrence and development of IS. Our finding increased our knowledge regarding the effect of *CYP4V2* gene on the process of IS, and also provided some data for further exploring the relationship between *CYP4V2* and IS risk in different populations, which will help to establish new warning and treatment methods for IS in future studies. In subsequent studies, we will further explore the functions of these SNPs based on this study, in order to provide new theoretical basis and targets for the diagnosis and treatment of IS.

## Conclusion

In conclusion, our results first provided evidence that *CYP4V2* rs1398007 might be a risk factor for IS after Bonferroni correction in the Chinese Han population, which provides instructive clues for *CYP4V2* polymorphisms in the pathogenesis of IS. However, the potential contribution in other populations remains to be determined.

## Supplementary Information

**Additional file 1**: **Table S1**. Primers sequence of PCR and UEP for* CYP4V2* SNPs in this study.** Table S2**.The information about* CYP4V2* SNPs and the association with IS susceptibility in allele model.** Table S3**. The potential functional SNPs in human* CYP4V2* gene

## Data Availability

The datasets generated and/or analysed during the current study are available in the zenodo repository (https://zenodo.org/record/6504233#.YmueVqyOND8).
