# Supplementary material for: CYP4 subfamily V member 2 (CYP4V2) polymorphisms were associated with ischemic stroke in Chinese Han population
Source: BMC Med Genomics. 2022 Nov 28;15:246. doi: 10.1186/s12920-022-01393-8 (PMC9703829; doi:10.1186/s12920-022-01393-8)
Supplement: Supplementary file 1 — Additional file 1: Table S1. Primers sequence of PCR and UEP for CYP4V2 SNPs in this study. Table S2.The information about CYP4V2 SNPs and the association with IS susceptibility in allele model. Table S3. The potential functional SNPs in human CYP4V2 gene [file 12920_2022_1393_MOESM1_ESM.docx]

**Suppl_Table 1. Primers sequence of PCR and UEP for *CYP4V2* SNPs in this study**

| **SNPs** | **First Primer(5'-3')** | **Second Primer (5'-3')** | **UEP SEQ (5'-3')** |
| --- | --- | --- | --- |
| rs1398007 | ACGTTGGATGGCGCAGGAACAGCCCCGTG | ACGTTGGATGACTTCCCCGCGCGGACCTG | ggCCCGGTCCCCGGAAC |
| rs13146272 | ACGTTGGATGTGGCTTTGGCTTGATCTCTG | ACGTTGGATGTCAGGGACTTACACTGTTGG | TGGTAAAAGTATGTAGGATCT |
| rs3736455 | ACGTTGGATGAGTCCAAACAGAAGCATGTG | ACGTTGGATGCTGCGTTTATTTTTGGAGGG | TCAAATTATACAGGTCATCGC |
| rs1053094 | ACGTTGGATGCAGATTTTATAACCTGGAGC | ACGTTGGATGGTGACTGGGATTCTATGAGG | ggtAAGACTATAAGTGCACGA |
| rs56413992 | ACGTTGGATGGAAAAAGTCCTTCCAAGATG | ACGTTGGATGCATGTAGGGTCACTAAAAAC | AAAACAAAATGTAAGGGATAAAT |

SNP: Single nucleotide polymorphism; UEP: Unextended mini sequencing primer; SEQ, sequence.

**Suppl_Table 2.The information about *CYP4V2* SNPs and the association with IS susceptibility in allele model**

| **SNPs ID** | **Chr: Position** | **Alleles** | **Frequency (MAF)** | | **Function** | **Protein residue** | **O(HET)** | **E(HET)** | ***p*-value**  **for HWE** | **Call rate** | **OR (95% CI)** | ***p*** |
| --- | --- | --- | --- | --- | --- | --- | --- | --- | --- | --- | --- | --- |
|  |  | (minor/major) | Case | Control |  |  |  |  |  |  |  |  |
| rs1398007 | 4:186191678 | T/C | 0.320 | 0.310 | 5' UTR |  | 0.435 | 0.428 | 0.770 | 100.0% | 1.05 (0.88–1.25) | 0.590 |
| rs13146272 | 4:186199057 | A/C | 0.377 | 0.368 | missense | Gln259Lys | 0.468 | 0.465 | 0.929 | 99.7% | 1.04 (0.87–1.23) | 0.681 |
| rs3736455 | 4:186201165 | G/T | 0.404 | 0.384 | synonymous | Ala270= | 0.510 | 0.473 | 0.077 | 99.8% | 1.09 (0.92–1.29) | 0.317 |
| rs1053094 | 4:186211877 | A/T | 0.362 | 0.343 | 3' UTR |  | 0.459 | 0.451 | 0.711 | 99.7% | 1.09 (0.92–1.29) | 0.346 |
| rs56413992 | 4:186213040 | T/C | 0.209 | 0.199 | 3' UTR |  | 0.317 | 0.318 | 0.896 | 99.7% | 1.07 (0.87–1.31) | 0.534 |

SNP: single nucleotide polymorphism; IS; ischemic stroke; MAF: minor allele frequency; O(HET): Observed heterozygotes; E(HET): Expected heterozygotes; HWE: Hardy-Weinberg equilibrium.

**Suppl_Table 3. The potential functional SNPs in human *CYP4V2* gene**

| rs | Chr:Position | Allele | SNPinfo Web Server | | | | | Haploreg | AFR freq | AMR freq | ASN freq | EUR freq |
| --- | --- | --- | --- | --- | --- | --- | --- | --- | --- | --- | --- | --- |
|  |  |  | TFBS | Splicing | miRNA(miRanda) | nsSNP | Polyphen |  |  |  |  |  |
| rs1398007 | 4:187349826 | T/C | Y | -- | -- | -- | -- | Promoter histone marks, DNAse, Proteins bound, Motifs changed, Selected eQTL hits | 0.23 | 0.44 | 0.29 | 0.53 |
| rs13146272 | 4:187357205 | A/C | -- | Y | -- | Y | benign | Motifs changed, GRASP QTL hits, Selected eQTL hits | 0.60 | 0.51 | 0.37 | 0.66 |
| rs3736455 | 4:187359313 | G/T | -- | Y | -- | -- | -- | Enhancer histone marks, GRASP QTL hits, Selected eQTL hits | 0.59 | 0.53 | 0.36 | 0.68 |
| rs1053094 | 4:187370025 | A/T | -- | -- | Y | -- | -- | Motifs changed, Selected eQTL hits | 0.64 | 0.52 | 0.34 | 0.53 |
| rs56413992 | 4:187371188 | C/T | -- | -- | Y | -- | -- | Enhancer histone marks, Motifs changed, Selected eQTL hits | 0.11 | 0.21 | 0.19 | 0.17 |

TFBS, transcription factor binding sites; QTL, quantitative trait locus; AFR, African; AMR, American; ASN, Asian; EUR, European.

SNPinfo Web Server: https://snpinfo.niehs.nih.gov/snpinfo/index.html.

HaploReg v4.1: https://pubs.broadinstitute.org/mammals/haploreg/haploreg.php.
